# Inhibition of Poly (ADP-Ribose) Glycohydrolase Accelerates Osteoblast Differentiation in Preosteoblastic MC3T3-E1 Cells

**DOI:** 10.3390/ijms23095041

**Published:** 2022-05-02

**Authors:** Yuka Sasaki, Ryusuke Nakatsuka, Takuma Inouchi, Mitsuko Masutani, Tadashige Nozaki

**Affiliations:** 1 Department of Pharmacology, Faculty of Dentistry, Osaka Dental University, Osaka 573-1121, Japan; nakatsuka-r@cc.osaka-dent.ac.jp (R.N.); inouchi-t@cc.osaka-dent.ac.jp (T.I.); nozaki@cc.osaka-dent.ac.jp (T.N.); 2 Department of Molecular and Genomic Biomedicine, Center for Bioinformatics and Molecular Medicine, Nagasaki University Graduate School of Biomedical Sciences, Nagasaki 852-8523, Japan; mmasutan@nagasaki-u.ac.jp

**Keywords:** PARP1, PARG, poly ADP-ribosylation, osteoblast differentiation, olaparib, PDD00017273

## Abstract

Poly ADP-ribosylation (PARylation) is a post-translational modification catalyzed by poly (ADP-ribose) polymerase (PARP) family proteins such as PARP1. Although PARylation regulates important biological phenomena such as DNA repair, chromatin regulation, and cell death, little is known about the relationship between osteoblast differentiation and the PARylation cycle involving PARP1 and the poly (ADP-ribose)-degrading enzyme poly (ADP-ribose) glycohydrolase (PARG). Here, we examined the effects of PARP inhibitor olaparib, an approved anti-cancer agent, and PARG inhibitor PDD00017273 on osteoblast differentiation. Olaparib decreased alkaline phosphatase (ALP) activity and suppressed mineralized nodule formation evaluated by Alizarin Red S staining in preosteoblastic MC3T3-E1 cells, while PDD00017273 promoted ALP activity and mineralization. Furthermore, PDD00017273 up-regulated the mRNA expression levels of osteocalcin and bone sialoprotein, as osteoblast differentiation markers, and osterix as transcription inducers for osteoblast differentiation, whereas olaparib down-regulated the expression of these genes. These findings suggest that PARG inhibition by PDD00017273 accelerates osteoblast differentiation in MC3T3-E1 cells. Thus, PARG inhibitor administration could provide therapeutic benefits for metabolic bone diseases such as osteoporosis.

## 1. Introduction

Bone mass is maintained by a balance between bone formation by osteoblasts and bone resorption by osteoclasts. An imbalance in bone homeostasis can lead to metabolic bone diseases such as osteoporosis. During the process of osteoblast differentiation from bone marrow mesenchymal stem cells (MSCs), several intracellular signaling pathways including the transforming growth factor (TGF)-β, bone metalloproteinase (BMP), and Wnt/β-catenin pathways are activated, and osteoblast cells secrete osteoblast differentiation markers in a stepwise manner, including alkaline phosphatase (ALP) at the early stage of differentiation and osteopontin (OPN), bone sialoprotein (BSP), and osteocalcin (OCN) at the late stage of differentiation and bone mineralization [1,2]. The expression levels of these marker genes are mainly regulated by runt-related transcription factor 2 (RUNX2), osterix (OSX), and activating transcription factor 4 (ATF4) during osteoblast differentiation and bone mineralization [1]. Recently, it was reported that post-translational modifications of proteins such as glycosylation, ubiquitination, and poly ADP-ribosylation (PARylation) may regulate the process of osteoblast differentiation [3,4,5]. Although osteoblast differentiation-related factors are well-understood, the relationships between post-translational modifications and osteoblast differentiation have not been clarified.

PARylation is catalyzed by poly (ADP-ribose) polymerase (PARP) family proteins such as PARP1 that transfer ADP-ribose units to their target proteins through NAD^+^ [6,7]. In this reaction, poly (ADP-ribose) (PAR) synthesized by PARPs is mainly degraded to ADP-ribose by poly (ADP-ribose) glycohydrolase (PARG) [8,9]. The PARylation cycle involving PARPs and PARG regulates various biological phenomena including DNA repair, chromatin regulation, cell death, and metabolism through the synthesis and degradation of PAR on target proteins [6,10]. A PARP inhibitor olaparib has been approved as a therapeutic agent for cancer patients with *BRCA1* or *BRCA2* mutations [11], although its effects on metabolic bone diseases and bone formation remain unclear. A previous study detected PAR in the calcification region of the bone matrix [12], suggesting that PARP1 is related to osteoblast differentiation. Furthermore, the PARP inhibitor PJ34 was shown to suppress osteoblast differentiation in murine MSCs [13]. However, it has not been clarified how alterations to intracellular PAR levels through regulation by PARP1 and PARG affect osteoblast differentiation.

Here, we examined the effects of olaparib and PDD00017273, a potent PARG inhibitor, on osteoblast differentiation in preosteoblastic MC3T3-E1 cells. Olaparib reduced ALP activity, suppressed calcium deposition when analyzed by Alizarin Red S staining, and down-regulated expression of osteoblast differentiation marker genes. Conversely, PDD00017273 accelerated osteoblast differentiation accompanied by an enhancement of ALP activity and upregulated expression of differentiation-related genes. These findings suggest that PARG inhibition promotes osteoblast differentiation, and that the development of PARG inhibitors for clinical use will be useful in therapeutic strategies for metabolic bone diseases.

## 2. Results

### 2.1. Effects of Olaparib and PDD00017273 on Cell Viability of MC3T3-E1 Cells

**Figure 1:**
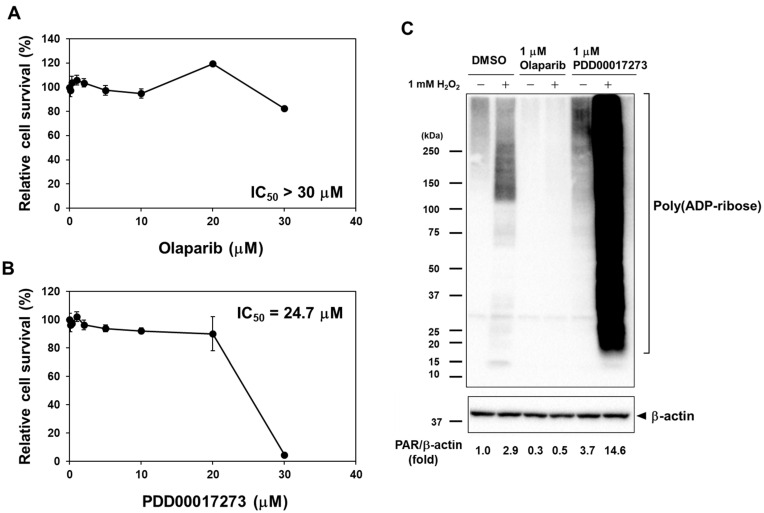
Effects of olaparib and PDD00017273 on cell cytotoxicity and PARylation activity in MC3T3-E1 cells. (**A**,**B**) Cell viability in olaparib- and PDD00017273-treated MC3T3-E1 cells. MC3T3-E1 cells were cultured in the condition of serum starvation for 24 h, and then treated with olaparib or PDD00017273 at concentrations of 0–30 µM in differentiation medium for 3 days. Cell viability was measured by CCK assay. (**C**) Intracellular PAR levels in MC3T3-E1 cells after treatment with olaparib and PDD00017273. MC3T3-E1 cells were treated with DMSO (solvent), 1 µM olaparib, or 1 µM PDD00017273 for 24 h, and then incubated in the absence or presence of 1 mM H_2_O_2_ for 10 min. Whole-cell extracts were analyzed by Western blotting using an anti-PAR antibody.

To evaluate the effects of PARP and PARG inhibitors on osteoblast differentiation, we first examined the cell viability of preosteoblastic MC3T3-E1 cells after treatment with olaparib and PDD00017273. As shown in Figure 1A,B, olaparib and PDD00017273 had little effect on the cell viability of MC3T3-E1 cells. The IC_50_ values for olaparib and PDD00017273 were >30 µM and 24.7 µM, respectively. To avoid cytotoxic effects, we used olaparib and PDD00017273 at a concentration of 1 µM in these experiments.

### 2.2. Evaluation of Intracellular PAR Levels in Olaparib- and PDD00017273-Treated MC3T3-E1 Cells

To determine whether intracellular PAR was regulated by olaparib and PDD00017273, we analyzed the PAR levels in olaparib- and PDD00017273-treated MC3T3-E1 cells by Western blot analysis using an anti-PAR antibody (Figure 1C). Briefly, the cells were treated with olaparib or PDD00017273 for 24 h, and then incubated in the presence or absence of hydrogen peroxide (H_2_O_2_) for 10 min to induce hyperactivation of PARPs by DNA damage. Olaparib induced 70% lower PAR level than DMSO treatment in MC3T3-E1 cells in the absence of H_2_O_2_. In contrast, PDD00017273 induced 14.6- and 3.7-fold higher levels of PAR accumulation in MC3T3-E1 cells with or without H_2_O_2_, respectively, suggesting that many target proteins were PARylated in the cells. These results suggested that olaparib and PDD00017273 inhibited the PARP and PARG enzymatic activities, respectively.

### 2.3. Effects of Olaparib and PDD00017273 on ALP Activity and Mineralization in MC3T3-E1 Cells

**Figure 2:**
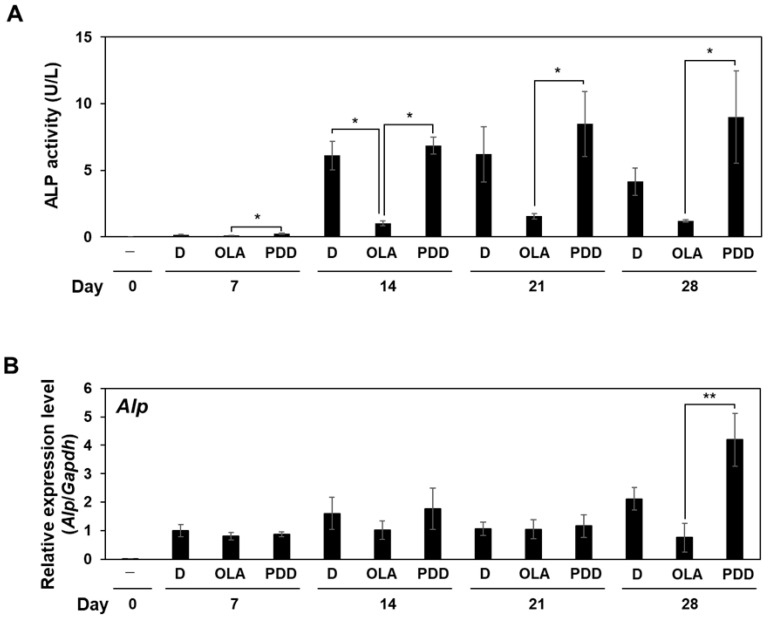
Effects of olaparib and PDD00017273 on ALP activity and mRNA expression of *Alp* during osteoblast differentiation. (**A**) ALP activity in olaparib- and PDD00017273-treated MC3T3-E1 cells. MC3T3-E1 cells were treated with DMSO, 1 µM olaparib, or 1 µM PDD00017273 after incubation in differentiation medium for 0–28 days. ALP activity was measured at specified time points. (**B**) Levels of *Alp* mRNA expression determined by qRT-PCR and normalized to the mRNA expression in DMSO-treated cells on day 7 after incubation in differentiation medium. *Alp* expression was increased in PDD00017273-treated cells compared with olaparib-treated cells at 28 days after incubation with differentiation medium. To obtain these data, at least three independent experiments were performed. D, DMSO; OLA, 1 µM olaparib; PDD, 1 µM PDD00017273. Data are shown as mean ± SE. * *p* < 0.05, ** *p* < 0.01 (Tukey’s test).

**Figure 3:**
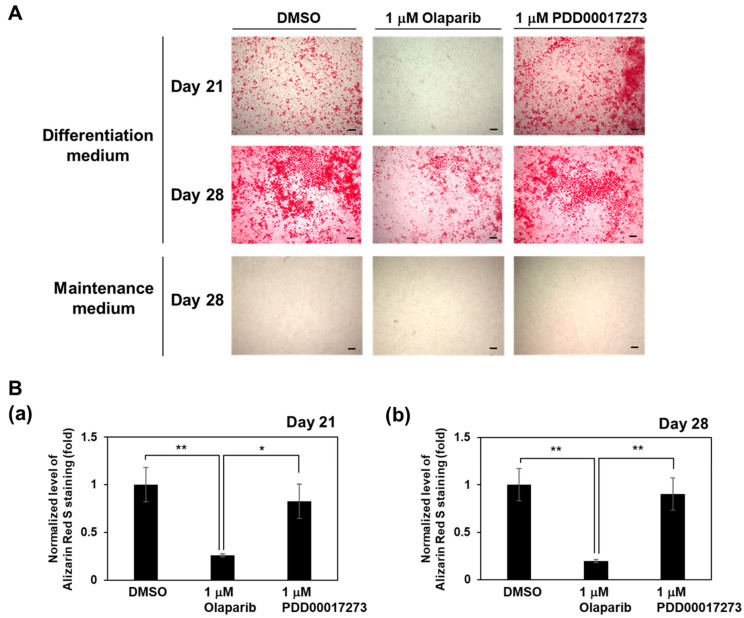
Effects of olaparib and PDD00017273 on matrix mineralization in MC3T3-E1 cells. MC3T3-E1 cells were cultured in differentiation medium in the presence of DMSO, 1 µM olaparib, or 1 µM PDD00017273 for 21 and 28 days after incubation of differentiation medium or maintenance medium. (**A**) Mineralized nodules were detected by Alizarin Red S staining. Scale bars indicate 200 μm. (**B**) Alizarin Red S staining level was quantified by measuring the absorbance at 415 nm. Graph showed quantification data of matrix mineralization at 21 days (**a**) and 28 days (**b**) after incubation in differentiation medium. In groups cultured in maintenance medium for 28 days, the absorbance at 415 nm was less than 0.1. Data are shown as mean ± SE. * *p* < 0.05, ** *p* < 0.01 (Tukey’s test).

To determine whether the alterations in intracellular PAR levels mediated by olaparib and PDD00017273 had effects on the osteoblast differentiation process, we examined the ALP activity after incubation of MC3T3-E1 cells in osteoblast differentiation medium. ALP is an osteoblast differentiation marker that hydrolyzes pyrophosphate to inorganic phosphate, thereby promoting mineralization [1]. As shown in Figure 2A, olaparib treatment decreased ALP activity at 14, 21, and 28 days compared with DMSO treatment in MC3T3-E1 cells. PDD00017273-treated cells showed a similar level of ALP activity as DMSO-treated cells from 7–21 days, and a higher ALP activity was noted at day 28 in PDD00017273-treated cells compared with DMSO control, although the difference was not statistically significant. The mRNA level of *Alp* was decreased in olaparib-treated cells compared with DMSO- and PDD00017273-treated cells at 28 days after incubation in differentiation medium (Figure 2B). We also observed that the mRNA level of *Alp* was increased only at day 28 in PDD00017273-treated cells compared with the DMSO-treated cells, although the difference was not statistically significant. To further assess the effect of olaparib and PDD00017273 on osteoblast differentiation induction, calcium phosphate deposits were visualized by Alizarin Red S staining. In accordance with the results for ALP activity, matrix mineralization determined by Alizarin Red S staining was significantly reduced in olaparib-treated cells compared with DMSO- and PDD00017273-treated cells at 21 and 28 days after incubation in differentiation medium (Figure 3). These results suggested that olaparib suppressed osteoblast differentiation, while PDD00017273 accelerated the differentiation.

### 2.4. The mRNA Expression of Osteoblast Differentiation Markers in Olaparib- and PDD0017273-Treated MC3T3-E1 Cells

**Figure 4:**
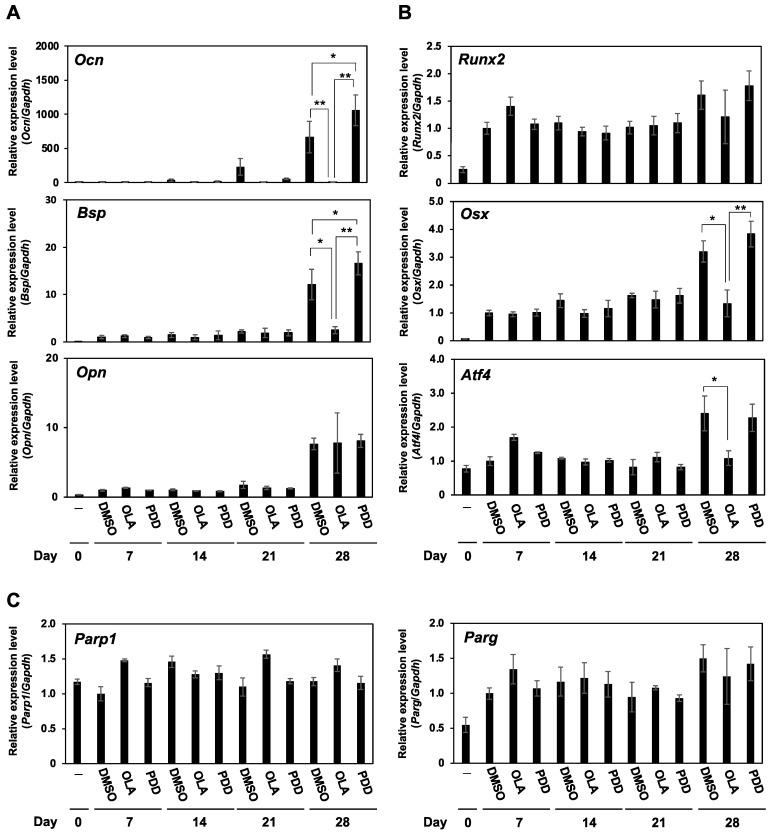
Olaparib downregulates and PDD00017273 upregulates the mRNA expression of osteogenic differentiation-related genes. MC3T3-E1 cells were treated with 1 µM olaparib, 1 µM PDD00017273, or DMSO for 0–28 days in differentiation medium. The mRNA expression levels of osteoblast differentiation-related genes were analyzed by qRT-PCR and normalized to the mRNA expression in DMSO-treated cells on day 7 after incubation in differentiation medium. (**A**) *Ocn*, *Bsp*, and *Opn*, as osteoblast differentiation markers. (**B**) *Runx2*, *Osx*, and *Atf4*, as transcription factors for osteoblast differentiation. (**C**) *Parp1* and *Parg*. OLA, 1 µM olaparib; PDD, 1 µM PDD00017273. Data are shown as mean ± SE. * *p* < 0.05, ** *p* < 0.01 (Tukey’s test).

To investigate the impacts of olaparib and PDD00017273 on the process of osteoblast differentiation, we analyzed the mRNA expression of osteoblast differentiation-related genes by qRT-PCR (Figure 4). The expression levels of *Ocn* and *Bsp* were down-regulated in olaparib-treated cells compared with DMSO- and PDD00017273-treated cells at 28 days after incubation in differentiation medium, while *Opn* expression was unchanged. Notably, PDD00017273-treated cells showed higher expression of *Ocn* (*p* < 0.05) and *Bsp* (*p* < 0.01) than DMSO-treated cells on day 28. Osteoblast differentiation is regulated by three essential transcription factors, *Runx2*, *Osx*, and *Atf4*. The mRNA expression levels of *Osx* and *Atf4*, as late-stage differentiation inducers, were significantly suppressed in olaparib-treated cells compared with DMSO- and/or PDD00017273-treated cells, while expression of *Runx2*, an early-stage differentiation inducer, was not changed (Figure 4B). Olaparib and PDD00017273 did not alter the mRNA expression levels of *Parp1* and *Parg*, as their target proteins, during osteoblast differentiation (Figure 4C). Taken together, these results suggested that increases in PARylation activity accelerated osteoblast differentiation in MC3T3-E1 cells. 

## 3. Discussion

Recently, PARylation was proposed as a novel regulatory mechanism for osteoblast differentiation. Briefly, PARP1 activation following stimulation with H_2_O_2_ promoted osteoblast differentiation in human MSCs and SAOS-2 cells [14,15], while the administration of PARP inhibitor PJ34 suppressed osteogenic differentiation accompanied by the downregulation of *Parp1* mRNA expression in murine MSCs [13]. However, little has been reported on the relationship between osteoblast differentiation and the PARylation cycle. Therefore, we evaluated the effects of the PARP inhibitor olaparib, as an approved anti-cancer agent, and the PARG inhibitor PDD00017273 on osteoblast differentiation. We found that olaparib downregulated the intracellular level of PAR and decreased ALP activity and bone mineralization assessed by Alizarin Red S staining in preosteoblastic MC3T3-E1 cells. These results are consistent with a previous report showing that PARP inhibition by PJ34 suppressed osteoblast differentiation [13]. PDD00017273 upregulated intracellular PAR, while the mRNA expression of *Parg* was not decreased. Notably, PDD00017273 increased ALP activity and mineralization. These results suggested that PDD00017273 inhibited the enzymatic activity of Parg without the downregulation of *Parg* mRNA in the differentiation condition, and promoted osteoblastogenesis.

Osteoblastogenesis is regulated by many cellular signaling pathways, including the Wnt/β-catenin, BMP, TGF-β, parathyroid hormone, and fibroblast growth factor pathways. In the downstream of these pathways, three essential transcription factors regulate the mRNA expression of osteoblast differentiation-related genes [1,2]. RUNX2 is an essential transcription factor that broadly regulates the expression of osteoblastogenic markers such as *ALP*, *BSP*, *OCN*, *OPN*, and *COL1A*, and mainly acts at the early stage of differentiation [1,16]. OSX and ATF4, as important transcription factors for osteoblast differentiation, act at the late stage of differentiation. OSX is also involved in the expression of *ALP*, *BSP*, *OCN*, and *OPN*, and ATF4 can induce the mRNA expression of *OCN*, *BSP*, and *COL1A* [1]. In addition, *OSX* expression was enhanced by RUNX2 [17] and PARP1 was identified as a positive regulator of RUNX2 expression via PARylation [18,19]. In the present study, we consider that *Runx2* is sufficiently upregulated in differentiation medium used by our study, and this could be the reason why *Runx2* expression was not further altered in olaparib and PDD00017273-treated cells, although Runx2-regulated genes including *Osx*, *Alp*, *Bsp,* and *Ocn* were downregulated in olaparib-treated cells. Meanwhile, PDD00017273-treated cells showed upregulated expression of *Ocn* and *Bsp* compared with DMSO- and olaparib-treated cells. Taken together, these results suggest that the increase of PARylated proteins by PDD00017273 could promote osteoblast differentiation after lineage commitment, and it is speculated that PDD00017273 can promote the PARylation of Runx2 and accelerate osteoblast differentiation. Recently, Muller *et al.* reported that PAR forms liquid droplets with calcium ions and promotes the physicochemical process of physiological extracellular matrix calcification [20]. In this study, PDD00017273 significantly accumulated intracellular PAR in MC3T3-E1 cells. Intracellular PAR itself may be involved in the promotion of the mineralization process, although it is necessary to analyze the relationship between PAR and bone mineralization in the process of osteoblast differentiation.

PARylation by PARP1 is well-known to be an important factor in the DNA damage response (DDR) [8]. Previous studies have shown that DDR-related proteins suppressed osteoblast differentiation. For example, deficiency of the early DDR proteins ATM and ERCC1, as nucleotide excision repair (NER) proteins, was associated with suppression of osteoblast differentiation [21,22]. Meanwhile, the early DDR marker γH2AX (phosphorylation of histone H2AX on serine 139) was detected in unmineralized and mineralized bone regions in the fetal sheep growth plate [20], suggesting that DNA damage is detected at a constant frequency during the differentiation process. PARP1 is also known to be an important DNA repair protein and involved in certain DNA repair pathways such as the base excision repair, non-homologous end-joining, and NER pathways [23]. In the present study, olaparib delayed osteoblast differentiation, suggesting that PARP1 inhibition by olaparib may induce the inactivation of DNA repair pathways during osteoblast differentiation. The regulatory mechanism for osteoblastogenesis by activated DDR pathways remains to be clarified, and further studies are warranted to determine the relationships between DDR proteins including PARP1 or PARP2 and osteoblast differentiation. 

Olaparib has been approved as a therapeutic agent for cancer patients harboring *BRCA1*/*BRCA2* mutations [11]. Meanwhile, a PARG inhibitor for clinical use has not been developed, even though PDD00017273 was shown to be a potent and specific PARG inhibitor [24]. The present study showed that the accumulation of intracellular PAR by PDD00017273 promoted osteoblast differentiation in MC3T3-E1 cells, suggesting that PARG inhibitors can activate the process of osteoblastogenesis, although we should accurately validate the clinical relevance of PDD00017273 by in vivo experiments. The development of novel PARG inhibitors for clinical use that can promote osteoblast differentiation will provide valuable therapeutic agents for metabolic bone diseases such as osteoporosis.

## 4. Materials and Methods

### 4.1. Cell Culture and Osteoblast Differentiation

Mouse preosteoblastic MC3T3-E1 cells were obtained from RIKEN BRC Cell Bank (Tsukuba, Japan). The cells were maintained in Minimum Essential Medium (no ascorbic acid) (Sigma-Aldrich, St. Louis, MO, USA) supplemented with 10% fetal bovine serum (FBS) (Gibco, Thermo Fisher Scientific, Tokyo, Japan) and 1% penicillin-streptomycin (Nacalai Tesque, Kyoto, Japan). To induce osteoblast differentiation, MC3T3-E1 cells were seeded on 6-, 48-, or 96-well plates at a density of 6.6 × 10^3^ cells/cm^2^ and cultured for 2 days in maintenance medium. The medium was then changed to differentiation medium containing 1% ascorbic acid, 0.2% hydrocortisone, 2% β-glycerophosphate, 10% FBS (Gibco), and 1% penicillin-streptomycin (Nacalai Tesque) in the presence of olaparib, PDD00017273, or dimethylsulfoxide (DMSO, solvent). The differentiation medium was replaced every 4 days, and the cells were allowed to differentiate for 0, 7, 14, 21, and 28 days for various experiments. The cells were cultured in a humidified atmosphere containing 5% CO_2_ at 37 °C. Olaparib was purchased from ChemScene (Monmouth Junction, NJ, USA), and PDD00017273 [24] was purchased from Cayman Chemical Company (Ann Arbor, MI, USA). 

### 4.2. Cell Proliferation Assay

MC3T3-E1 cells were seeded on 96-well plates at a density of 8 × 10^3^ cells/well. After 8 h of incubation, the medium was changed to serum-free medium to synchronize the same cell cycle phase. After 24 h, the cells began to culture in a differentiation medium containing olaparib, PDD00017273, or DMSO for 3 days. The cell viability of the treated cells was analyzed by CCK assay using Cell Counting Reagent SF (Nacalai Tesque) in accordance with the manufacturer’s instructions. 

### 4.3. ALP Activity Assay

The enzymatic activity of ALP was determined using a TRACP & ALP Assay Kit (TaKaRa Bio, Shiga, Japan) in accordance with the manufacturer’s protocols. Briefly, the cells were washed with saline and solubilized with 1% NP-40. The cell extracts were incubated in solution containing p-nitrophenyl phosphate at 37 °C. The reaction was stopped by the addition of 0.5 N NaOH, and the absorbance at 405 nm was measured with a microplate reader SpectraMax 50S (Molecular Devices Japan, Tokyo, Japan). A standard curve was constructed using calf intestinal ALP (TaKaRa Bio), and the ALP activity in the samples was calculated. One unit (U) of ALP activity represents the hydrolysis of 1 μmol p-nitrophenyl phosphate per minute at 37 °C.

### 4.4. Alizarin Red S Staining

The mineralization of MC3T3-E1 cells was determined by staining with Alizarin Red S, which binds to calcium phosphate and produces red staining. The cells were washed with phosphate-buffered saline and fixed with 10% neutral-buffered formalin solution for 15 min at room temperature. The cells were then incubated with Alizarin Red S solution (PG Research, Tokyo, Japan) for 30 min at room temperature. After washing with distilled water, 5% formic acid was added to the plates, and the absorbance at 415 nm was measured with a microplate reader SpectraMax 50S (Molecular Devices Japan).

### 4.5. Quantitative RT-PCR (qRT-PCR)

Total RNA was prepared from the cells using a High Pure RNA isolation kit (Roche, Basal, Switzerland). The total RNA was reverse-transcribed to cDNA using a High-Capacity cDNA Reverse Transcription Kit (Thermo Fisher Scientific). The cDNA was subjected to qRT-PCR using SYBR Green with a StepOnePlus Real-Time PCR System (Thermo Fisher Scientific) in accordance with the manufacturer’s instructions. The mouse primer sequences are listed in Table 1. 

### 4.6. Western Blot Analysis for PAR Detection

Western blotting was performed as described previously [25]. Briefly, whole-cell lysates from MC3T3-E1 cells were mixed with Laemmli’s buffer, separated by 5–20% SDS-polyacrylamide gel electrophoresis, and transferred onto PVDF membranes. The following primary antibodies were used: anti-PAR (Cell Signaling Technology, Danvers, MA, USA) and anti-β-actin (Sigma-Aldrich). Immune complexes were visualized using horseradish peroxidase-linked secondary antibodies (Cytiva, Tokyo, Japan). Protein bands were visualized with an enhanced chemiluminescence kit (Merck, Branchburg, NJ, USA). Image quantification was performed with Image J software (NIH, Rockville, MD, USA).

### 4.7. Statistical Analysis

Data were shown as mean ± standard error of the mean (SE). Data were analyzed using Tukey’s test and JASP 0.15 software (University of Amsterdam, Amsterdam, The Netherlands). Statistical significance was accepted for values of *p* < 0.05.

## Figures and Tables

**Table 1:** Primers for qRT-PCR.

Target Gene	Forward (5′–3′)	Reverse (5′–3′)
*Parp1*	GCAGCGAGAGTATTCCCAAG	CCGTCTTCTTGACCTTCTGC
*Parg*	CTGTTCACTGAGGTGCTGGA	TCTCAGGCACAAACTGATCG
*Alp*	AACCCAGACACAAGCATTCC	GAGAGCGAAGGGTCAGTCAG
*Atf4*	CGATGCTCTGTTTCGAATGGA	CCAACGTGGTCAAGAGCTCAT
*Bsp*	TTTATCCTCCTCTGAAACGGT	GTTTGAAGTCTCCTCTTCCTCC
*Ocn*	AAGCAGGAGGGCAATAAGGT	TTTGTAGGCGGTCTTCAAGC
*Opn*	AGCAAGAAACTCTTCCAAGCAA	GTGAGATTCGTCAGATTCATCCG
*Osx*	CTCGTCTGACTGCCTGCCTAG	GCGTGGATGCCTGCCTTGTA
*Runx2*	CGCACGACAACCGCACCAT	CAGCACGGAGCACAGGAAGTT
*Gapdh*	TGGTGAAGGTCGGTGTGAAC	AGGGGTCGTTGATGGCAACA

## Data Availability

Not applicable.
